# Interface crack between different orthotropic media under uniform heat flow

**DOI:** 10.1186/s40064-016-3105-5

**Published:** 2016-09-06

**Authors:** Sheng-Hu Ding, Xing Li

**Affiliations:** School of Mathematics and Computer Science, Ningxia University, Yinchuan, 750021 China

**Keywords:** Functionally graded orthotropic media, Interface zone, Crack, Singular integral equation, Stress intensity factors

## Abstract

In this paper, plane thermo-elastic solutions are presented for the problem of a crack in two bonded homogeneous orthotropic media with a graded interfacial zone. The graded interfacial zone is treated as a nonhomogeneous interlayer having spatially varying thermo-elastic moduli between dissimilar, homogeneous orthotropic half-planes, which is assumed to vary exponentially in the direction perpendicular to the crack surface. Using singular integral equation method, the mixed boundary value conditions with respect to the temperature field and those with respect to the stress field are reduced to a system of singular integral equations and solved numerically. Numerical results are obtained to show the influence of non-homogeneity parameters of the material thermo-elastic properties, the orthotropy parameters and the dimensionless thermal resistance on the temperature distribution and the thermal stress intensity factors.

## Background

Functionally graded materials (FGMs) are designed as the special materials which have changed micro-structure and mechanical/thermal properties in the space to meet the required functional performance (Niino et al. 1987; Suresh and Mortensen 1998). The advantages of FGMs are that the magnitude of residual and thermal stresses can be reduced, and the bonding strength and fracture toughness of such materials can be improved. From both the phenomenological and mechanistic viewpoints, the tailoring capability to produce gradual changes of thermo-physical properties in the spatial domain is the key point for the impressive progress in the areas of functionally graded materials (Miyamoto et al. 1999).

By introducing the concept of the FGMs, extensive research on all aspects of fracture of isotropic and orthotropic FGMs under mechanical or thermal loads has been considered (Choi et al. 1998; Choi 2003; Wang et al. 2004; El-Borgi and Hidri 2006; Han and Wang 2006; Cheng et al. 2010; Ding and Li 2014; Kim and Paulino 2002; Dag 2006; Zhou et al. 2007). By considering changes in both elastic and thermal properties, Jin and Noda (1991) studied the transient thermo-elastic problems of functionally graded material with a crack. Fujimoto and Noda (2001) investigated the thermal cracking under a transient-temperature field in a ceramic/metal functionally graded plate. In addition, assuming the surfaces of the crack are insulated, thermal stresses around a crack in the interfacial layer between two dissimilar elastic half-planes are studied by Itou (2004). With the introduction of the thermal resistance concept, the thermal stress intensity factors for the interface crack between functionally graded layered structures under the thermal loading are investigated by Ding and Li (2015). Zhou and Lee (2011) studied the thermal fracture problem of a functionally graded coating-substrate structure of finite thickness with a partially insulated interface crack subjected to thermal–mechanical supply. Chen (2005) obtained the thermal stress intensity factors (TSIFS) of a graded orthotropic coating-substrate structure with an interface crack. Zhou et al. (2010) considered the thermal response of an orthotropic functionally graded coating-substrate structure with a partially insulated interface crack.

Using mesh-free model, Dai et al. (2005) studied the active shape control as well as the dynamic response repression of the functionally graded material (FGM) plate containing distributed piezoelectric sensors and actuators. Natarajan et al. (2011) considered the linear free flexural vibration of cracked functionally graded material plates by using the extended finite element method. Using extended finite element method, fatigue crack growth simulations of bi-material interfacial cracks have been considered under thermo-elastic loading (Pathak et al. 2013). Using element free Galerkin method, Pathak et al. (2014) studied quasi-static fatigue crack growth simulations of homogeneous and bi-material interfacial cracks under mechanical as well as thermo-elastic load.

Layered FGM structure are very import in practical engineering (Sofiyev and Avcar 2010; Sofiyev et al. 2012; Ding et al. 2014; Ding et al. 2015). The research of thermal elastic crack problem in layered structure is helpful for the design and application of functionally graded materials. This paper explores the thermal–mechanical response of layered and graded structures using the integral equation approach. The analytical results of the cracked layered material systems with the material properties in the graded coating varying as an exponential function has been obtained by using the integral transform technique. The surface of the crack is assumed to be part of the thermal insulation. The temperature distributions along the crack line are presented. The TSIFS under thermo-mechanical loadings are obtained, which is very important for the designing of layered orthotropic media.

## Problem formulation

**Fig. 1:**
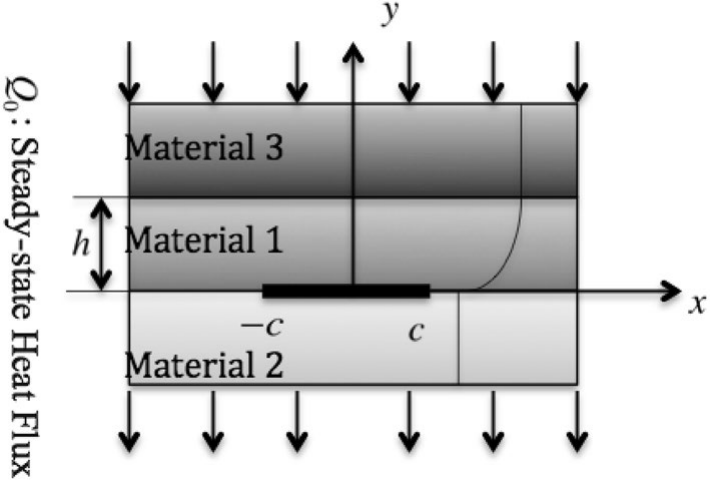
Geometry of the layered orthotropic media under steady-state heat flows

As shown in Fig. 1, the problem under consideration consists of a functionally graded orthotropic strip (FGOS) of thickness *h* bonded to two homogeneous semi-infinite orthotropic media with a partially insulated interface crack of length 2*c* along the *x*-axis is considered. The subscript *j*(*j* = 1, 2, 3) indicates the FGOS and two semi-infinite orthotropic media respectively. The remaining thermo-mechanical properties depend on the *y*-coordinate only and are modeled by an exponential function

1 $$ \left( {k_{x}^{(1)} ,k_{y}^{(1)} } \right) = \left( {k_{x}^{(2)} ,k_{y}^{(2)} } \right)\exp \left( {{{\delta y} \mathord{\left/ {\vphantom {{\delta y} c}} \right. \kern-0pt} c}} \right) $$

2 $$ \left( {k_{x}^{(3)} ,k_{y}^{(3)} } \right) = \left( {k_{x}^{(2)} ,k_{y}^{(2)} } \right)\exp \left( {{{\delta h} \mathord{\left/ {\vphantom {{\delta h} c}} \right. \kern-0pt} c}} \right) $$

where $k_{x}^{(2)} ,k_{y}^{(2)}$ are the thermal conductivities for the homogeneous orthotropic substrate *II*, and *δ* is an arbitrary nonzero constant.

The temperature satisfies

3 $$ \frac{\partial }{\partial x}\left( {k_{x}^{(j)} \frac{{\partial T_{j} }}{\partial x}} \right) + \frac{\partial }{\partial y}\left( {k_{y}^{(j)} \frac{{\partial T_{j} }}{\partial y}} \right) = 0\quad (j = 1 - 3) $$

Substituting Eqs. (1) and (2) into the Eq. (3), the heat equation can be given by

4 $$ k_{xy0} \frac{{\partial^{2} T_{1} }}{{\partial x^{2} }} + \delta \frac{{\partial T_{1} }}{\partial y} + \frac{{\partial^{2} T_{1} }}{{\partial y^{2} }} = 0\quad 0 < y < h $$

5 $$ k_{xy0} \frac{{\partial^{2} T_{j} }}{{\partial x^{2} }} + \frac{{\partial^{2} T_{j} }}{{\partial y^{2} }} = 0\quad (j = 2,3) $$

where $$ k_{xy0} = k_{x}^{(2)} /k_{y}^{(2)} $$.

The heat flux components are written as

6 $$ \begin{aligned} k_{3} \frac{{\partial T_{1} (x,y)}}{\partial y} = - Q_{0} ,y \to + \infty ,\left| x \right| < + \infty \hfill \\ k_{2} \frac{{\partial T_{2} (x,y)}}{\partial y} = - Q_{0} ,y \to - \infty ,\left| x \right| < + \infty \hfill \\ \end{aligned} $$

We define the following dimensionless quantities

7 $$ \left\{ {\begin{array}{*{20}l} {\left( {\overline{x} ,\overline{y} ,\overline{h} } \right) = {{\left( {x,y,h} \right)} \mathord{\left/{\vphantom{{\left( {x,y,h} \right)} {c,\overline{T}_{j} = {{T_{j} } \mathord{\left/ {\vphantom {{T_{j} } {\left( {{{- Q_{0} c} \mathord{\left/{\vphantom {{- Q_{0} c} {k_{y}^{(2)} }}} \right. \kern-0pt} {k_{y}^{(2)} }}} \right), \, }}} \right. \kern-0pt} {\left({{{- Q_{0} c} \mathord{\left/ {\vphantom {{- Q_{0} c} {k_{y}^{(2)}}}} \right. \kern-0pt} {k_{y}^{(2)} }}} \right),\, }}}}} \right. \kern-0pt} {c,\overline{T}_{j} = {{T_{j} } \mathord{\left/ {\vphantom {{T_{j} } {\left( {{{ - Q_{0} c} \mathord{\left/ {\vphantom {{- Q_{0} c} {k_{y}^{(2)} }}} \right. \kern-0pt} {k_{y}^{(2)} }}} \right), \, }}} \right. \kern-0pt} {\left( {{{ - Q_{0} c} \mathord{\left/ {\vphantom {{ - Q_{0} c} {k_{y}^{(2)} }}} \right. \kern-0pt} {k_{y}^{(2)} }}} \right), \, }}}}} \hfill & \quad {j = 1 - 3} \hfill \\ {\overline{\sigma }_{jkl} = {{\sigma_{jkl} } \mathord{\left/ {\vphantom {{\sigma_{jkl} } {\left( {{{ - E_{0} Q_{0} \alpha_{2} c} \mathord{\left/ {\vphantom {{ - E_{0} Q_{0} \alpha_{2} c} {k_{y}^{(2)} }}} \right. \kern-0pt} {k_{y}^{(2)} }}} \right)}}} \right. \kern-0pt} {\left( {{{ - E_{0} Q_{0} \alpha_{2} c} \mathord{\left/ {\vphantom {{ - E_{0} Q_{0} \alpha_{2} c} {k_{y}^{(2)} }}} \right. \kern-0pt} {k_{y}^{(2)} }}} \right)}},} \hfill & \quad {(k,l = x,y)} \hfill \\ {\left( {\overline{u}_{j} ,\overline{v}_{j} } \right) = {{\left( {u_{j} ,v_{j} } \right)} \mathord{\left/ {\vphantom {{\left( {u_{j} ,v_{j} } \right)} {\left( {{{ - Q_{0} \alpha_{2} c^{2} } \mathord{\left/ {\vphantom {{ - Q_{0} \alpha_{2} c^{2} } {k_{y}^{(2)} }}} \right. \kern-0pt} {k_{y}^{(2)} }}} \right)}}} \right. \kern-0pt} {\left( {{{ - Q_{0} \alpha_{2} c^{2} } \mathord{\left/ {\vphantom {{ - Q_{0} \alpha_{2} c^{2} } {k_{y}^{(2)} }}} \right. \kern-0pt} {k_{y}^{(2)} }}} \right)}}} \hfill & {} \hfill \\ \end{array} } \right. $$

8 $$ \left\{ {\begin{array}{*{20}l} {\left( {\overline{\alpha }_{ij}^{(1)} ,\overline{\alpha }_{ij}^{(2)} ,\overline{\alpha }_{ij}^{(3)} } \right) = \frac{{\left( {\alpha_{ij}^{(1)} ,\alpha_{ij}^{(2)} ,\alpha_{ij}^{(3)} } \right)}}{{\alpha_{0} }},} \hfill & \quad {(i,j = x,y)} \hfill \\ {\left( {\overline{E}_{xx}^{0} ,\overline{E}_{yy}^{0} } \right) = \frac{{\left( {E_{xx}^{0} ,E_{yy}^{0} } \right)}}{{E_{0} }}} \hfill & {} \hfill \\ {\left( {\overline{C}_{ij}^{(1)} ,\overline{G}_{66}^{(1)} ,\overline{C}_{ij}^{(2)} ,\overline{G}_{66}^{(2)} ,\overline{C}_{ij}^{(3)} ,\overline{G}_{66}^{(3)} } \right) = \frac{{\left( {C_{ij}^{(1)} ,G_{66}^{(1)} ,C_{ij}^{(2)} ,G_{66}^{(2)} ,C_{ij}^{(3)} ,G_{66}^{(3)} } \right)}}{{E_{0} }},} \hfill & \quad {(i,j = 1,2)} \hfill \\ \end{array} } \right. $$

where *α*_0_ and *E*_0_ are the typical values of the coefficient of linear thermal expansion and the Young’s modulus of elasticity for the homogeneous orthotropic substrate, respectively. But for simplicity, in what follows, the bar appearing with the dimensionless quantities is omitted.

The Duhamel–Neumann constitutive equations for the plane thermo-elastic problem are given by Nowinski (1978)

9 $$ \sigma_{xx} = C_{11} \frac{{\partial u_{{}} }}{\partial x} + C_{12} \frac{{\partial v_{{}} }}{\partial y} - \theta_{1} T,\quad \sigma_{yy} = C_{12} \frac{{\partial u_{{}} }}{\partial x} + C_{22} \frac{\partial v}{\partial y} - \theta_{2} T, \quad \sigma_{xy} = C_{66} \left( {\frac{\partial u}{\partial y} + \frac{\partial v}{\partial x}} \right) $$

in which

10 $$ \theta_{1}^{{}} = C_{11}^{{}} \alpha_{xx}^{{}} + C_{12}^{{}} \alpha_{yy}^{{}} ,\quad \theta_{2}^{{}} = C_{12}^{{}} \alpha_{xx}^{{}} + C_{22}^{{}} \alpha_{yy}^{{}} ,\quad C_{66}^{{}} = G_{xy}^{{}} $$

The elastic stiffness coefficients and the coefficients of the linear thermal expansion in dimensionless form are modeled to take the following forms

11 $$ \left\{ {\begin{array}{*{20}l} {\left( {C_{11}^{(1)} ,C_{12}^{(1)} ,C_{22}^{(1)} ,C_{66}^{(1)} } \right) = \left( {C_{11}^{(2)} ,C_{12}^{(2)} ,C_{22}^{(2)} ,C_{66}^{(2)} } \right)\exp (\beta y)} \hfill \\ {\left( {\alpha_{xx}^{(1)} ,\alpha_{yy}^{(1)} } \right) = \left( {\alpha_{xx}^{(2)} ,\alpha_{yy}^{(2)} } \right)\exp (\gamma y)} \hfill \\ \end{array} } \right. $$

where superscripts 1, 2 refer to the FGOS and the homogeneous orthotropic substrate *II*, respectively, *β* and *γ* are graded parameters. The properties of material 3 can be found in Eq. (11) when *y* is taken as *h*. In Eq. (11), elastic stiffness coefficients in dimensionless form can be represented by the Young’s moduli and the Poisson’s ratios as

12 $$ C_{11}^{(2)} = \frac{{E_{xx}^{(2)} }}{{1 - \nu_{yx} \nu_{xy} }},\quad C_{22}^{(2)} = \frac{{E_{yy}^{(2)} }}{{1 - \nu_{yx} \nu_{xy} }},\quad C_{12}^{(2)} = \frac{{E_{yy}^{(2)} \nu_{xy} }}{{1 - \nu_{yx} \nu_{xy} }} $$

where *ν*_*ij*_ are the Poisson’s ratios and assumed to be constant. $E_{xx}^{(2)}$ and $E_{yy}^{(2)}$ are Young’s moduli for the homogeneous orthotropic substrate *II*, respectively.

Substituting Eq. (9) into the equations of equilibrium for the forces reduces these equations to the forms

13 $$ \left\{ {\begin{array}{*{20}l} {C_{11}^{(2)} \frac{{\partial^{2} u_{1} }}{{\partial x^{2} }} + C_{66}^{(2)} \frac{{\partial^{2} u_{1} }}{{\partial y^{2} }} + \left( {C_{12}^{(2)} + C_{66}^{(2)} } \right)\frac{{\partial^{2} v_{1} }}{\partial x\partial y} + \beta C_{66}^{(2)} \left( {\frac{{\partial u_{1} }}{\partial y} + \frac{{\partial v_{1} }}{\partial x}} \right) = \theta_{1}^{(2)} e^{\gamma y} \frac{{\partial T_{1} }}{\partial x}} \hfill \\ {C_{22}^{(2)} \frac{{\partial^{2} v_{1} }}{{\partial y^{2} }} + C_{66}^{(2)} \frac{{\partial^{2} v_{1} }}{{\partial x^{2} }} + \left( {C_{12}^{(2)} + C_{66}^{(2)} } \right)\frac{{\partial^{2} u_{1} }}{\partial x\partial y} + \beta \left( {C_{12}^{(2)} \frac{{\partial u_{1} }}{\partial x} + C_{22}^{(2)} \frac{{\partial v_{1} }}{\partial y}} \right) = \theta_{2}^{(2)} e^{\gamma y} \left[ {(\beta + \gamma )T_{1} + \frac{{\partial T_{1} }}{\partial y}} \right]} \hfill \\ \end{array} } \right. $$

14 $$ \left\{ \begin{aligned} C_{11}^{(2)} \frac{{\partial^{2} u_{2} }}{{\partial x^{2} }} + C_{66}^{(2)} \frac{{\partial^{2} u_{2} }}{{\partial y^{2} }} + \left( {C_{12}^{(2)} + C_{66}^{(2)} } \right)\frac{{\partial^{2} v_{2} }}{\partial x\partial y} = \theta_{1}^{(2)} \frac{{\partial T_{2} }}{\partial x} \hfill \\ C_{22}^{(2)} \frac{{\partial^{2} v_{2} }}{{\partial y^{2} }} + C_{66}^{(2)} \frac{{\partial^{2} v_{2} }}{{\partial x^{2} }} + \left( {C_{12}^{(2)} + C_{66}^{(2)} } \right)\frac{{\partial^{2} u_{2} }}{\partial x\partial y} = \theta_{2}^{(2)} \frac{{\partial T_{2} }}{\partial y} \hfill \\ \end{aligned} \right. $$

15 $$ \left\{ \begin{aligned} C_{11}^{(2)} \frac{{\partial^{2} u_{3} }}{{\partial x^{2} }} + C_{66}^{(2)} \frac{{\partial^{2} u_{3} }}{{\partial y^{2} }} + \left( {C_{12}^{(2)} + C_{66}^{(2)} } \right)\frac{{\partial^{2} v_{3} }}{\partial x\partial y} = \theta_{1}^{(2)} e^{\gamma h} \frac{{\partial T_{3} }}{\partial x} \hfill \\ C_{22}^{(2)} \frac{{\partial^{2} v_{3} }}{{\partial y^{2} }} + C_{66}^{(2)} \frac{{\partial^{2} v_{3} }}{{\partial x^{2} }} + \left( {C_{12}^{(2)} + C_{66}^{(2)} } \right)\frac{{\partial^{2} u_{3} }}{\partial x\partial y} = \theta_{2}^{(2)} e^{\gamma h} \frac{{\partial T_{3} }}{\partial y} \hfill \\ \end{aligned} \right. $$

## Boundary conditions

The temperature filed can be provided using the following boundary condition

16 $$ T_{1} (x,y) = T_{3} (x,y) \quad |x| < + \infty , \, y = h $$

17 $$ \frac{{\partial T_{1} (x,y)}}{\partial y} = \left\{ {\begin{array}{*{20}l} { - Bi\left( {T_{1} (x,y) - T_{2} (x,y)} \right)} \hfill & {\left| x \right| \le c,} \hfill & {y = 0} \hfill \\ {\frac{{\partial T_{2} (x,y)}}{\partial y}} \hfill & {\left| x \right| < + \infty ,} \hfill & {y = 0} \hfill \\ {\frac{{\partial T_{3} (x,y)}}{\partial y}} \hfill & {\left| x \right| < + \infty ,} \hfill & {y = h} \hfill \\ { - Q_{0} /k_{y}^{(3)} } \hfill & {y \to + \infty ,} \hfill & {|x| < + \infty } \hfill \\ \end{array} } \right. $$

where $Bi = 1/k_{y}^{(1)} (0)/R_{c}$ is dimensionless thermal resistance through the crack region. *R*_*c*_ is the thermal resistance through the crack region.

The boundary conditions of the stress and displacement field can be given by

18 $$ \sigma_{1xy} (x,y) = \left\{ {\begin{array}{*{20}c} {0\quad y = 0,} &\quad {\left| x \right| \le c} \\ {\sigma_{3xy} (x,y)} &\quad {y = h} \\ \end{array} } \right.,\quad \sigma_{1yy} (x,y) = \left\{ {\begin{array}{*{20}c} {0\quad y = 0,} &\quad {\left| x \right| \le c} \\ {\sigma_{3xy} (x,y)} &\quad {y = h} \\ \end{array} } \right., $$

19 $$ u_{1} (x,h^{ - } ) = u_{3} (x,h^{ + } )\quad v_{1} (x,h^{ - } ) = v_{3} (x,h^{ + } )\quad \left| x \right| < \infty $$

## Heat conduction problem

By using Fourier transform, the solutions of Eqs. (4) and (5) are given by

20 $$ \left\{ {\begin{array}{*{20}l} {T_{1} (x,y) = \int_{ - \infty }^{ + \infty } {(M_{1} (\omega )\exp (s_{1} y) + } M_{2} (\omega )\exp (s_{2} y))\exp ( - i\omega x)d\omega + \frac{{1 - e^{ - \delta y} }}{\delta },} \hfill & {0 < y < h} \hfill \\ {T_{2} (x,y) = \int_{ - \infty }^{ + \infty } {(M_{3} (\omega )\exp (p_{1} y) + } M_{4} (\omega )\exp (p_{2} y))\exp ( - i\omega x)d\omega + y,} \hfill & { \, y \le 0} \hfill \\ {T_{3} (x,y) = \int_{ - \infty }^{ + \infty } {(M_{5} (\omega )\exp (o_{1} y) + } M_{6} (\omega )\exp (o_{2} y))\exp ( - i\omega x)d\omega + e^{ - \delta h} y + 1/\delta - \frac{1 + \delta h}{\delta }e^{ - \delta h} ,} \hfill & {y \ge h} \hfill \\ \end{array} } \right. $$

where $M_{k} (\omega )(k = 1 - 6)$ can be found in “Appendix 1”. $s_{k} ,p_{k}$ and $o_{k}$ are the roots of the characteristic polynomials, which can be given by

21 $$ s_{1,2} = \frac{1}{2}\left( { - \delta \pm \sqrt {\delta^{2} + 4k_{xy0} \omega^{2} } } \right),\quad p_{1,2} = \pm \sqrt {k_{xy0} } \left| \omega \right|,\quad o_{1,2} = \pm \sqrt {k_{xy0} } \left| \omega \right| $$

Introducing the unknown density function

22 $$ \phi (x) = \frac{\partial }{\partial x}\left[ {T_{1} (x,0^{ + } ) - T_{2} (x,0^{ - } )} \right] $$

From (17), we obtain

23 $$ \int_{ - 1}^{ + 1} {\left( {\frac{1}{u - x} + H(x,u)} \right)\phi (u)du = \frac{ - 2\pi }{{\sqrt {k_{xy0} } }}} $$

where the kernel $H(x,u)$ can be found in “Appendix 1”.

## Thermal stress analysis

By using the standard Fourier transforms to Eqs. (13)–(15), following results for the displacement fields for the FGOS and two homogeneous orthotropic media are obtained

24 $$ \left\{ {\begin{array}{*{20}l} {u_{1} (x,y) = \int_{ - \infty }^{ + \infty } {\left( {\sum\limits_{j = 1}^{4} {C_{j} (\omega )e^{{m_{j} y}} } } \right)e^{ - ix\omega } d\omega } + \int_{ - \infty }^{ + \infty } {\left( {\sum\limits_{j = 1}^{2} {\frac{{\xi_{j} (\omega )}}{{d_{j} (\omega )}}e^{{(\gamma + s_{j} )y}} } } \right)e^{ - ix\omega } d\omega } } \hfill \\ {v_{1} (x,y) = \int_{ - \infty }^{ + \infty } {\left( {\sum\limits_{j = 1}^{4} {C_{j} (\omega )q_{j} (\omega )e^{{m_{j} y}} } } \right)e^{ - ix\omega } d\omega } + \int_{ - \infty }^{ + \infty } {\left( {\sum\limits_{j = 1}^{2} {\frac{{\xi_{j + 2} (\omega )}}{{d_{j} (\omega )}}e^{{(\gamma + s_{j} )y}} } } \right)e^{ - ix\omega } d\omega + \chi_{1} e^{\gamma y} + \chi_{2} e^{(\gamma - \delta )y} } } \hfill \\ \end{array} } \right. $$

25 $$ \left\{ {\begin{array}{*{20}l} {u_{2} (x,y) = \int_{ - \infty }^{ + \infty } {\left( {C_{5} (\omega )e^{{n_{1} y}} + C_{6} (\omega )e^{{n_{2} y}} + \frac{{\xi_{5} (\omega )}}{{d_{3} (\omega )}}e^{{(\gamma + p_{1} )y}} } \right)} e^{ - i\omega x} d\omega } \hfill \\ {v_{2} (x,y) = \int_{ - \infty }^{ + \infty } {\left( {C_{5} (\omega )q_{5} (\omega )e^{{n_{1} y}} + C_{6} (\omega )q_{6} (\omega )e^{{n_{2} y}} + \frac{{\xi_{6} (\omega )}}{{d_{3} (\omega )}}e^{{(\gamma + p_{1} )y}} } \right)} e^{ - i\omega x} d\omega + \frac{{\theta_{2}^{(2)} }}{{2C_{12}^{(2)} }}y^{2} } \hfill \\ \end{array} } \right. $$

26 $$ \left\{ {\begin{array}{*{20}l} {u_{3} (x,y) = \int_{ - \infty }^{ + \infty } {\left( {C_{7} (\omega )e^{{n_{3} y}} + C_{8} (\omega )e^{{n_{4} y}} + \frac{{\xi_{7} (\omega )}}{{d_{4} (\omega )}}e^{{(\gamma + o_{2} )y}} } \right)} e^{ - i\omega x} d\omega } \hfill \\ {v_{3} (x,y) = \int_{ - \infty }^{ + \infty } {\left( {C_{7} (\omega )q_{7} (\omega )e^{{n_{3} y}} + C_{8} (\omega )q_{8} (\omega )e^{{n_{4} y}} + \frac{{\xi_{8} (\omega )}}{{d_{4} (\omega )}}e^{{(\gamma + o_{2} )y}} } \right)} e^{ - i\omega x} d\omega + \chi_{1} e^{\gamma h} + \chi_{2} e^{(\gamma - \delta )h} } \hfill \\ \end{array} } \right. $$

where $C_{j} (\omega )(j = 1 - 8)$ are unknown function. $\xi_{j} ,q_{j} (j = 1 - 8)$, $d_{j} (j = 1 - 4)$, $\chi_{j} (j = 1,2)$ are given in “Appendix 1”. $m_{j} (j = 1 - 4)$ and $n_{j} (j = 1 - 4)$ are the roots of the characteristic polynomials, which can be given by

27 $$ m_{1,2} = \frac{1}{2}\left( { - \beta - \sqrt {\beta^{2} - 2\Delta_{1} \pm 2\sqrt {(\Delta_{1} )^{2} - 4\Delta_{2} } } } \right) $$

28 $$ m_{3,4} = \frac{1}{2}\left( { - \beta + \sqrt {\beta^{2} - 2\Delta_{1} \pm 2\sqrt {(\Delta_{1} )^{2} - 4\Delta_{2} } } } \right) $$

29 $$ n_{1,2} = - \frac{1}{2}\sqrt { - 2\Delta_{3} \pm 2\sqrt {(\Delta_{3} )^{2} - 4\Delta_{4} } } ,\quad n_{3,4} = \frac{1}{2}\sqrt { - 2\Delta_{3} \pm 2\sqrt {(\Delta_{3} )^{2} - 4\Delta_{4} } } $$

where

$$ \Delta_{1} = \omega^{2} \left( {\frac{{(C_{12}^{(2)} )^{2} }}{{C_{22}^{(2)} C_{66}^{(2)} }} - \frac{{C_{11}^{(2)} }}{{C_{66}^{(2)} }} + 2\frac{{C_{12}^{(2)} }}{{C_{22}^{(2)} }}} \right),\quad \Delta_{2} = \omega^{4} \frac{{C_{11}^{(2)} }}{{C_{22}^{(2)} }} + \omega^{2} \beta^{2} \frac{{C_{12}^{(2)} }}{{C_{22}^{(2)} }},\quad \Delta_{3} = \Delta_{1} ,\quad \Delta_{4} = \omega^{4} \frac{{C_{11}^{2} }}{{C_{22}^{2} }} $$

## Solution procedure and near-tip field intensity factors

Introducing the density functions

30 $$ \varPhi_{1} (x) = \frac{\partial }{\partial x}\left[ {u(x,0^{ + } ) - u(x,0^{ - } )} \right],\quad \varPhi_{2} (x) = \frac{\partial }{\partial x}\left[ {v(x,0^{ + } ) - v(x,0^{ - } )} \right] $$

Substituting Eqs. (24)–(26) into Eqs. (18)–(19), we obtain

31 $$ \left\{ \begin{aligned} \int_{ - 1}^{1} {\left\{ {\left[ {\frac{1}{u - x} + K_{11} (x,u)} \right]\varPhi_{1} (u) + K_{12} (x,u)\varPhi_{2} (u)} \right\}} du = \frac{{2\pi \cdot \omega_{1}^{T} (x)}}{{\sqrt {k_{xy0} } }} \hfill \\ \int_{ - 1}^{1} {\left\{ {K_{21} (x,u)\varPhi_{1} (u) + \left[ {\frac{1}{u - x} + K_{22} (x,u)} \right]\varPhi_{2} (u)} \right\}} du = \frac{{2\pi \cdot \omega_{2}^{T} (x)}}{{\sqrt {k_{xy0} } }} \hfill \\ \end{aligned} \right. $$

where $K_{ij} (x,u)(i,j = 1,2),\;\omega_{1} (x)^{T} ,\omega_{2} (x)^{T}$ are given in “Appendix 2”.

The singular integral Eq. (31) are solved numerically with the unknown density functions $R_{1} (u)$ and $R_{2} (u)$ having the following form

32 $$ \left\{ {\begin{array}{*{20}c} {\varPhi_{1} (u) = \frac{{R_{1} (u)}}{{\sqrt {1 - u^{2} } }}} &\quad  {R_{1} (u) = \sum\limits_{n = 1}^{N} {b_{n} } T_{n} (u)} \\ {\varPhi_{2} (u) = \frac{{R_{2} (u)}}{{\sqrt {1 - u^{2} } }}} &\quad {R_{2} (u) = \sum\limits_{n = 1}^{N} {c_{n} } T_{n} (u)} \\ \end{array} } \right. $$

Once $R_{1} (u)$ and $R_{2} (u)$ have been determined, the thermal stress intensity factors ahead of the crack tip can be defined and calculated as follows

33 $$ \left\{ \begin{array}{l} K_{I} (1) = \mathop {\lim }\limits_{{x \to 1^{ + } }} \sqrt {2(x - 1)} \sigma_{yy} (x,0) = - \frac{{\sqrt {k_{xy0} } }}{2}R_{2} (1), \, \hfill \\ K_{I} ( - 1) = \mathop {\lim }\limits_{{x \to - 1^{ - } }} \sqrt {2( - x - 1)} \sigma_{yy} (x,0) = \frac{{\sqrt {k_{xy0} } }}{2}R_{2} ( - 1), \hfill \\ K_{II} (1) = \mathop {\lim }\limits_{{x \to 1^{ + } }} \sqrt {2(x - 1)} \sigma_{xy} (x,0) = - \frac{{\sqrt {k_{xy0} } }}{2}R_{1} (1), \, \hfill \\ K_{II} ( - 1) = \mathop {\lim }\limits_{{x \to - 1^{ - } }} \sqrt {2( - x - 1)} \sigma_{xy} (x,0) = \frac{{\sqrt {k_{xy0} } }}{2}R_{1} ( - 1). \hfill \\ \end{array} \right. $$

## Numerical results and discussion

In this paper, the orthotropy and non-homogeneity parameters of Tyrannohex can be found in Ootao and Tanigawa (2005). The material properties can be given by

$$ \begin{aligned} E_{xx} & = 135\,{\text{GPa,}}\quad E_{yy} = 87\,{\text{GPa,}}\quad\nu_{xy} = 0.15,\quad\nu_{yx} = 0.09667,\quad\alpha_{xx} = 0.32 \times 10^{ - 5} /^{ \circ } {\text{C}}, \\ \alpha_{yy} & = 0.32 \times 10^{ - 5} /^{ \circ } {\text{C}},\quad k_{x} = 2.81\;{\text{W/m}}\;^{ \circ } {\text{C}},\quad k_{y} = 3.08\;{\text{W/m}}\;^{ \circ } {\text{C}} \\ \end{aligned} $$

In the presented results the values of the thermal stress intensity factors are normalized by $k_{0} = E_{2} Q_{0} \alpha_{2} \sqrt c /k_{y}^{(2)}$. The crack is located along the interval $- 1 \, \le x \le \, 1$.

**Fig. 2:**
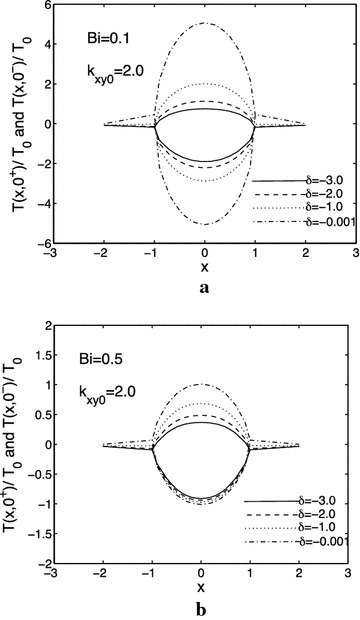
Influences of thermal conductivity parameter $$ \delta $$ on the normalized crack surfaces and crack extend line $$ y = 0 $$ temperatures $$ T(x, \, 0^{ + } )/T_{0} $$ and $$ T(x, \, 0^{ - } )/T_{0} , $$ $$ T_{0} = Q_{0} c/k_{y}^{(2)} , $$ $$ h = 1.0,k_{xy0} = 2.0, $$ **a** Bi = 0.1, **b** Bi = 0.5

Figure 2a, b show the effects of the thermal conductivity parameter $\delta$ on the crack surface temperature when $Bi = 0.1$ and $Bi = 0.5$, respectively. From Fig. 2a, b, it can be found that the temperature jump across the crack surfaces increases with an decrease of the absolute values of $\delta$. At the other hand, for smaller value of $Bi$, the temperature will become more pronounced. As expected, the temperature jump across the crack becomes more pronounced as the crack surfaces become more insulated, that is, as $Bi$ decreases.

**Fig. 3:**
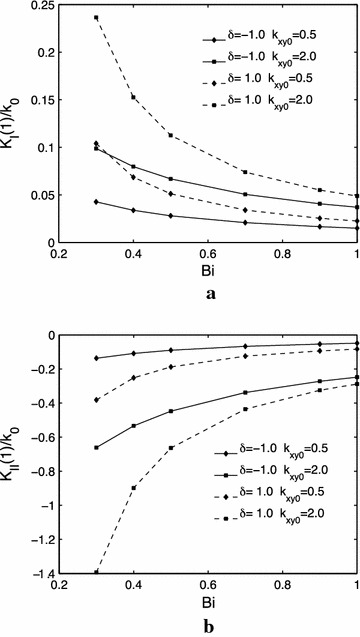
Influences of the thermal conductivity parameter $\delta$ and $k_{xy0}$ on the normalized thermal stress intensity factors, $h = 1.0$. **a** mode $I$. **b** mode $II$

Figure 3a, b show the effects of the thermal conductivity parameter $$ \delta $$ and $$ k_{xy0} $$ on the mode $$ I $$ and $$ k_{xy0} = 0.5 $$$$ II $$ thermal stress intensity factors. It can be found that the mode $$ I $$ thermal stress intensity factors increases with an increase of the thermal conductivity parameter $$ \delta $$ for either or $$ k_{xy0} = 2.0 $$; while increases with an increase of $$ k_{xy0} $$ for both $$ \delta = - 1.0 $$ and $$ \delta = 1.0 $$. And the values of mode $$ II $$ thermal stress intensity factors decreases with the increasing of the thermal conductivity parameter $$ \delta $$ regardless of the value of $$ k_{xy0} $$. Meanwhile, the values of mode $$ II $$ thermal stress intensity factors decreases with the increasing of an increase of $$ k_{xy0} $$ regardless of the value o $$ \alpha_{xx}^{(2)} $$ f $$ \delta $$.

**Fig. 4:**
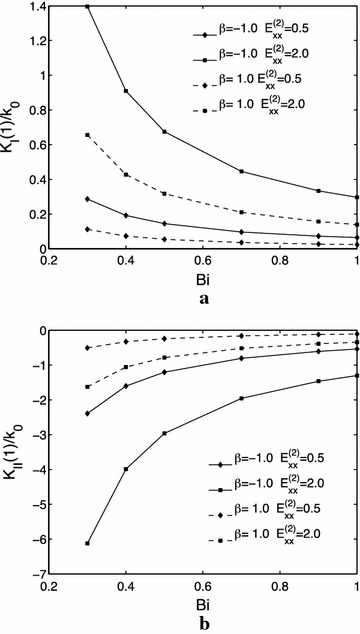
Influences of the stiffness parameter $\beta$ and $E_{xx}^{(2)}$ on the normalized mode thermal stress intensity factors. **a** $I$. **b** mode $II$

Figure 4a, b illustrate the effects of the stiffness parameter $\beta$ and $E_{xx}^{(2)}$ on the mode $I$ and $II$ thermal stress intensity factors. It can be seen that the mode $I$ thermal stress intensity factors increases with a decrease of the stiffness parameter $\beta$ for both $E_{xx}^{(2)} = 0.5$ and $E_{xx}^{(2)} = 2.0$; while increases with an increase of $E_{xx}^{(2)}$ regardless of the value of the stiffness parameter $\beta$. For the mode $II$ thermal stress intensity factors, the contrary is the case.

**Fig. 5:**
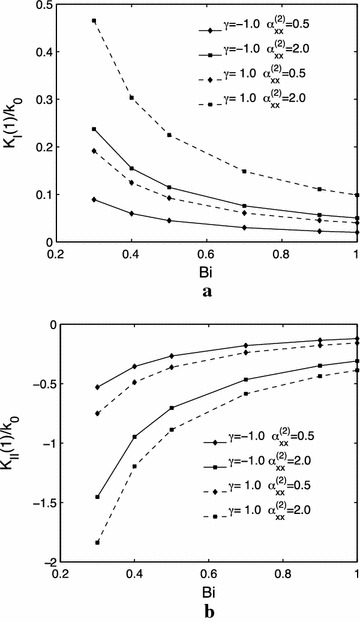
Influences of the thermal expansion parameter $\gamma$ and $\alpha_{xx}^{(2)}$ on the normalized mode thermal stress intensity factors. **a** $I$. **b** $II$

Figure 5a, b show the $II$ effects of the thermal expansion parameter $\gamma$ and on the mode $I$ and $II$ thermal stress intensity factors. It may be obtained that the absolute values of both mode $I$ and mode $II$ thermal stress intensity factors increases with an increase of the thermal expansion parameter $\gamma$ for either $k_{xy0} = 0.5$ or $k_{xy0} = 2.0$; and the absolute values of both mode $I$ and mode $II$ thermal stress intensity factors increases with an increase of $\alpha_{xx}^{(2)}$.

**Fig. 6:**
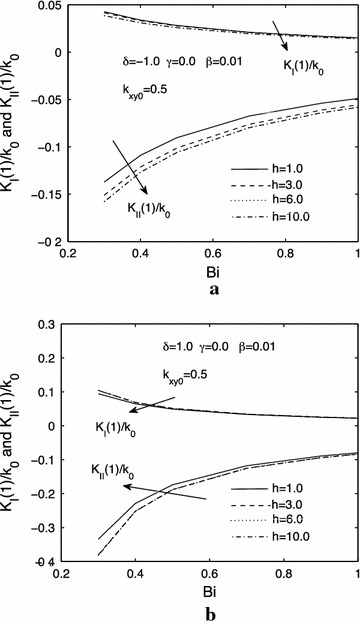
Influences of the thickness *h* of the functionally graded orthotropic strip on the normalized mode *I* and mode *II* thermal stress intensity factors, **a** *δ* = −1.0, **b** *δ* = 1.0

Figure 6a, b illustrate the effects of different thickness of functionally graded orthotropic strip on the mode $I$ and $II$ thermal stress intensity factors when $\delta = - 1.0$ and $\delta = 1.0$, respectively. We can see that the mode $I$ and thermal stress intensity factors increase or decrease with the increasing of $h$, and then reach a steady value.

## Conclusions

In this paper, thermo-mechanical stress and displacement fields for an interface crack between an orthotropic functionally graded interlayer and two homogeneous orthotropic media are obtained. In addition to the mechanical fields, temperature fields are also developed for exponentially varying thermal properties along the gradation direction. TSIFS are numerically calculated based on a singular integral equation derived from the dislocation density along the crack faces. The variations in temperature distribution and the thermal stress intensity factors due to the change in non-homogeneity parameters of the material thermo-elastic properties, the orthotropy parameters and the dimensionless thermal resistance are investigated.

## Appendix 1

The expressions of $M_{j} (w)(j = 1 - 6)$ are given by

34 $$ \left\{ {\begin{array}{*{20}l} {M_{1} (\omega ) = \frac{{ip_{1} (o_{2} - s_{2} )e^{{s_{2} h}} }}{{2\pi \omega ((s_{1} - o_{2} )(p_{1} - s_{2} )e^{{s_{1} h}} + (o_{2} - s_{2} )(p_{1} - s_{1} )e^{{s_{2} h}} )}}\int_{ - 1}^{1} \phi (x)e^{i\omega x} dx} \hfill \\ {M_{2} (\omega ) = \frac{{ip_{1} (s_{1} - o_{2} )e^{{s_{1} h}} }}{{2\pi \omega ((s_{1} - o_{2} )(p_{1} - s_{2} )e^{{s_{1} h}} + (o_{2} - s_{2} )(p_{1} - s_{1} )e^{{s_{2} h}} )}}\int_{ - 1}^{1} \phi (x)e^{i\omega x} dx} \hfill \\ {M_{3} (\omega ) = \frac{{i[s_{1} (o_{2} - s_{2} )e^{{s_{2} h}} + s_{2} (s_{1} - o_{2} )e^{{s_{1} h}} ]}}{{2\pi \omega ((s_{1} - o_{2} )(p_{1} - s_{2} )e^{{s_{1} h}} + (o_{2} - s_{2} )(p_{1} - s_{1} )e^{{s_{2} h}} )}}\int_{ - 1}^{1} \phi (x)e^{i\omega x} dx} \hfill \\ {M_{4} (\omega ) = M_{5} (\omega ) = 0} \hfill \\ {M_{6} (\omega ) = \frac{{ip_{1} (s_{1} - s_{2} )e^{{(s_{1} + s_{2} - o_{2} )h}} }}{{2\pi \omega ((s_{1} - o_{2} )(p_{1} - s_{2} )e^{{s_{1} h}} + (o_{2} - s_{2} )(p_{1} - s_{1} )e^{{s_{2} h}} )}}\int_{ - 1}^{1} \phi (x)e^{i\omega x} dx} \hfill \\ \end{array} } \right. $$

The kernel function $H(x,u)$ is

35 $$ H(x,u) = \mathop \int \nolimits_{0}^{ + \infty } \frac{ - 2}{{\sqrt {k_{xy0} } \omega }}\left[ {\frac{{s_{1} p_{1} (o_{2} - s_{2} )e^{{s_{2} h}} + s_{2} p_{1} (s_{1} - o_{2} )e^{{s_{1} h}} }}{{(s_{1} - o_{2} )(p_{1} - s_{2} )e^{{s_{1} h}} + (o_{2} - s_{2} )(p_{1} - s_{1} )e^{{s_{2} h}} }} + Bi - 1} \right]\sin [\omega (u - x)]d\omega $$

The expressions of $\xi_{j} ,q_{j} (j = 1 - 8)$, $d_{j} (j = 1 - 4)$, $\chi_{j} (j = 1,2)$ are given by

36 $$ \xi_{j} = i\omega M_{j} \left[ {\left( {\gamma + s_{j} } \right)\left( {\gamma + s_{j} + \beta } \right)\left( {\theta_{2}^{2} C_{12}^{2} - \theta_{1}^{2} C_{22}^{2} } \right)} \right] + C_{66}^{2} \left[ {\omega^{2} \theta_{1}^{2} + \left( {\gamma + s_{j} + \beta } \right)^{2} \theta_{2}^{2} } \right],\quad j = 1,2 $$

37 $$ \xi_{j} = \omega^{2} M_{j - 2} \left\{ {\left[ {\left( {\gamma + s_{j - 2} + \beta } \right)\left( {\theta_{1}^{2} C_{12}^{2} - \theta_{2}^{2} C_{11}^{2} } \right)} \right] + \left( {\gamma + s_{j - 2} } \right)C_{66}^{2} \left[ {\omega^{2} \theta_{1}^{2} + \left( {\gamma + s_{j - 2} + \beta } \right)^{2} \theta_{2}^{2} } \right]} \right\}\quad j = 3,4 $$

38 $$ \xi_{5} = i\omega M_{3} \left[ {p_{1}^{2} \left( {\theta_{2}^{2} C_{12}^{2} - \theta_{1}^{2} C_{22}^{2} } \right)} \right] + C_{66}^{2} \left[ {\omega^{2} \theta_{1}^{2} + p_{1}^{2} \theta_{2}^{2} } \right] $$

39 $$ \xi_{6} = \omega^{2} M_{3} \left[ {p_{1} \left( {\theta_{1}^{2} C_{12}^{2} - \theta_{2}^{2} C_{22}^{2} } \right)} \right] + p_{1} C_{66}^{2} \left[ {\omega^{2} \theta_{1}^{2} + p_{1}^{2} \theta_{2}^{2} } \right] $$

40 $$ \xi_{7} = i\omega M_{6} \left[ {o_{2}^{2} \left( {\theta_{2}^{2} C_{12}^{2} - \theta_{1}^{2} C_{22}^{2} } \right)} \right] + C_{66}^{2} \left[ {\omega^{2} \theta_{1}^{2} + o_{2}^{2} \theta_{2}^{2} } \right] $$

41 $$ \xi_{8} = \omega^{2} M_{6} \left[ {o_{2} \left( {\theta_{1}^{2} C_{12}^{2} - \theta_{2}^{2} C_{11}^{2} } \right)} \right] + o_{2} C_{66}^{2} \left[ {\omega^{2} \theta_{1}^{2} + o_{2}^{2} \theta_{2}^{2} } \right] $$

42 $$ q_{j} (\omega ) = \frac{{i\omega \left[ {m_{j} \left( {C_{12}^{2} + C_{66}^{2} } \right) + \beta C_{12}^{2} } \right]}}{{m_{j} (m_{j} + \beta )C_{22}^{2} - \omega^{2} C_{66}^{2} }},\quad j = 1 - 4 $$

43 $$ q_{j} (\omega ) = \frac{{i\omega n_{j - 4} \left( {C_{12}^{2} + C_{66}^{2} } \right)}}{{n_{j - 4}^{2} C_{22}^{2} - \omega^{2} C_{66}^{2} }},\quad j = 5 - 8 $$

44 $$ \begin{aligned} d_{j} (\omega ) & = C_{66}^{2} C_{12}^{2} [C_{11}^{2} \omega^{4} + \omega^{2} [(\gamma + s_{j} )^{2} + (\gamma + s_{j} + \beta )^{2} ] + (\gamma + s_{j} )^{2} \\ & \quad (\gamma + s_{j} + \beta )^{2} C_{22}^{2} ] + \omega^{2} (\gamma + s_{j} )(\gamma + s_{j} + \beta )[(C_{12}^{2} )^{2} - C_{11}^{2} C_{22}^{2} ],\quad j = 1 - 2 \\ \end{aligned} $$

45 $$ d_{3} (\omega ) = C_{66}^{2} \left[ {C_{11}^{2} \omega^{2} + 2\omega^{2} p_{1}^{2} C_{12}^{2} + p_{1}^{4} C_{22}^{2} } \right] + \omega^{2} p_{1}^{2} \left[ {\left( {C_{12}^{2} } \right)^{2} - C_{11}^{2} C_{22}^{2} } \right] $$

46 $$ d_{4} (\omega ) = C_{66}^{2} \left[ {C_{11}^{2} \omega^{2} + 2\omega^{2} o_{2}^{2} C_{12}^{2} + o_{2}^{4} C_{22}^{2} } \right] + \omega^{2} o_{2}^{2} \left[ {\left( {C_{12}^{2} } \right)^{2} - C_{11}^{2} C_{22}^{2} } \right] $$

47 $$ \chi_{1} = \frac{{\theta_{2}^{2} }}{{\delta C_{22}^{2} \gamma }}\quad \chi_{2} = - \frac{{\theta_{2}^{2} }}{{\delta C_{22}^{2} (\gamma - \delta )}} $$

## Appendix 2

The expressions of $K_{ij} (x,u)(i,j = 1,2)$,$\omega_{1} (x)^{T} ,\omega_{2} (x)^{T}$ are given by

48 $$ K_{11} (x,u) = \mathop {\lim }\limits_{{y \to 0^{ - } }} \mathop \int \nolimits_{0}^{ + \infty } \left( {\frac{2}{{\omega \sqrt {k_{xy0} } }}\left( {\frac{{A_{22} D_{16} }}{D}e^{{n_{2} y}} - \frac{{A_{21} D_{15} }}{D}e^{{n_{1} y}} } \right) - 1} \right)\sin [\omega (u - x)]d\omega $$

49 $$ K_{12} (x,u) = \mathop {\lim }\limits_{{y \to 0^{ - } }} \mathop \int \nolimits_{0}^{ + \infty } \frac{2i}{{\omega \sqrt {k_{xy0} } }}\left( {\frac{{A_{22} D_{26} }}{D}e^{{n_{2} y}} - \frac{{A_{21} D_{25} }}{D}e^{{n_{1} y}} } \right)\cos [\omega (u - x)]d\omega $$

50 $$ K_{21} (x,u) = \mathop {\lim }\limits_{{y \to 0^{ - } }} \mathop \int \nolimits_{0}^{ + \infty } \frac{2i}{{\omega \sqrt {k_{xy0} } }}\left( {\frac{{A_{24} D_{16} }}{D}e^{{n_{2} y}} - \frac{{A_{23} D_{15} }}{D}e^{{n_{1} y}} } \right)\cos [\omega (u - x)]d\omega $$

51 $$ K_{22} (x,u) = \mathop {\lim }\limits_{{y \to 0^{ - } }} \mathop \int \nolimits_{0}^{ + \infty } \left( {\frac{2}{{\omega \sqrt {k_{xy0} } }}\left( {\frac{{A_{24} D_{16} }}{D}e^{{n_{2} y}} - \frac{{A_{23} D_{15} }}{D}e^{{n_{1} y}} } \right) - 1} \right)\sin [\omega (u - x)]d\omega $$

52 $$ \left\{ {\begin{array}{*{20}l} {\omega_{1}^{T} (x) = \mathop \int \nolimits_{ - \infty }^{ + \infty } \left( {\sum\limits_{j = 1}^{8} {I_{j} J_{1j} - B_{23} e^{ - i\omega x} } } \right)dx} \hfill \\ {\omega_{2}^{T} (x) = \mathop \int \nolimits_{ - \infty }^{ + \infty } \left( {\sum\limits_{j = 1}^{8} {I_{j} J_{2j} - B_{21} - B_{22} } } \right)e^{ - i\omega x} dx} \hfill \\ \end{array} } \right. $$

with

53 $$ I_{1} = \xi_{5} /d_{3} - \mathop \sum \limits_{j = 1}^{2} \xi_{j} /d_{j} \quad I_{2} = \xi_{6} /d_{3} - \mathop \sum \limits_{j = 1}^{2} \xi_{j + 2} /d_{j} $$

54 $$ I_{3} = e^{{(\gamma + o_{2} )h}} q_{7} /d_{4} - \mathop \sum \limits_{j = 1}^{2} e^{{(\gamma + s_{j} )h}} \xi_{j} /d_{j} \quad I_{4} = e^{{(\gamma + o_{2} )h}} q_{8} /d_{4} - \mathop \sum \limits_{j = 1}^{2} e^{{(\gamma + s_{j} )h}} \xi_{j + 2} /d_{j} $$

55 $$ I_{5} = B_{23} - \mathop \sum \limits_{j = 1}^{2} F_{1j} \quad I_{6} = B_{21} + B_{22} - \mathop \sum \limits_{j = 1}^{2} B_{1j} $$

56 $$ I_{7} = e^{{(\gamma + o_{2} )h}} B_{31} + e^{{o_{2} h}} B_{32} - \mathop \sum \limits_{j = 1}^{2} e^{{(\gamma + s_{j} )h}} B_{1j} \quad I_{8} = e^{{(\gamma + o_{2} )h}} B_{33} - \mathop \sum \limits_{j = 1}^{2} e^{{(\gamma + s_{j} )h}} F_{1j} $$

57 $$ J_{1j} = ( - 1)^{j + 1} \left( {\frac{{A_{22} D_{j6} }}{D} - \frac{{A_{21} D_{j5} }}{D}} \right)\quad J_{2j} = ( - 1)^{j + 1} \left( {\frac{{A_{24} D_{j6} }}{D} - \frac{{A_{23} D_{j5} }}{D}} \right) $$

58 $$ A_{1j} = ( - i\omega )C_{12}^{2} + C_{22}^{2} m_{j} q_{j} \quad j = 1 - 4 $$

59 $$ B_{1j} = \left[ {( - i\omega )C_{12}^{2} \xi_{j} + C_{22}^{2} (\gamma + s_{j} )\xi_{j + 2} } \right]/d_{j} - \theta_{2}^{(2)} M_{j} \quad E_{1j} = C_{66}^{2} (m_{j} - i\omega q_{j} ) $$

60 $$ F_{1j} = C_{66}^{2} \left[ {(\gamma + s_{j} )\xi_{j} - i\omega \xi_{j + 2} } \right]/d_{j} \quad j = 1 - 4 $$

61 $$ A_{21} = C_{22}^{2} n_{1} q_{5} - i\omega C_{12}^{2} \quad A_{22} = C_{22}^{2} n_{2} q_{6} - i\omega C_{12}^{2} $$

62 $$ B_{21} = \left[ {C_{22}^{2} (\gamma + p_{1} )\xi_{6} - i\omega C_{12}^{2} } \right]/d_{3} \quad B_{22} = - \theta_{2}^{2} M_{3} $$

63 $$ A_{23} = C_{66}^{2} (n_{1} - i\omega q_{5} )\quad A_{24} = C_{66}^{2} (n_{2} - i\omega q_{6} ) $$

64 $$ B_{23} = C_{66}^{2} \left[ {(\gamma + p_{1} )\xi_{5} - i\omega \xi_{6} } \right]/d_{3} \quad A_{31} = C_{22}^{2} n_{3} q_{7} - i\omega C_{12}^{2} $$

65 $$ A_{32} = C_{22}^{2} n_{4} q_{8} - i\omega C_{12}^{2} \quad B_{31} = \left[ {C_{22}^{2} (\gamma + o_{2} )\xi_{8} - i\omega C_{12}^{2} \xi_{7} } \right]/d_{4} $$

66 $$ B_{32} = - \theta_{2}^{2} M_{6} e^{\gamma h} \quad A_{33} = C_{66}^{2} (n_{3} - i\omega q_{7} ) $$

67 $$ A_{34} = C_{66}^{2} (n_{4} - i\omega q_{8} )\quad B_{33} = C_{66}^{2} \left[ {(\gamma + o_{2} )\xi_{7} - i\omega \xi_{8} } \right]/d_{4} $$

Here $D$ is the determinant of the $D_{ij} (i,j = 1 - 8)$. $D_{ij}$ is the sub-determinant of the linear system of Eqs. (24)–(26) corresponding to the elimination of the $ith$ row and *j*th column.
